# Promoter Hyper-methylation of ZNF662 Restrains its Tumor Suppressing Function in Triple-Negative Breast Cancer Through Regulating NGF Signaling Axis

**DOI:** 10.7150/ijbs.102940

**Published:** 2025-06-12

**Authors:** Renjie Yu, Xi Peng, Zhaobo Cheng, Yue Wu, Huan Rong, Lin Yi, Jing Ran, Chaoqun Deng, Xiangyi Zhou, Ruijie Ming, Ningning Zhang, Xiaoyu Liu, Xiaohua Zeng, Yun Xiao, Xue Wang, Yongzhong Wu, Bo Qin, Tingxiu Xiang

**Affiliations:** 1 Department of Infectious Diseases, Chongqing Key Laboratory of Infectious Diseases and Parasitic Diseases, The First Affiliated Hospital of Chongqing Medical University, Chongqing, 400016, China.; 2 Chongqing Key Laboratory of Translational Research for Cancer Metastasis and Individualized Treatment, Chongqing University Cancer Hospital, Chongqing, 400030, China.; 3 Department of Oncology and Hematology, Chongqing Hospital of Traditional Chinese Medicine, Chongqing, China.; 4 The People's Hospital of Tongnan District Chongqing City, Chongqing, 402660, China.

**Keywords:** ZNF662, NGF, transcription factor, triple-negative breast cancer, methylation

## Abstract

Triple-negative breast cancer (TNBC) has the highest mortality rate among common cancers in women. Thus, the identification of new therapeutic targets is of major significance. Our study identifies ZNF662 as a novel member of KRAB-containing zinc finger proteins (KRAB-ZFPs) family in TNBC. However, its biological function and potential mechanisms in the progression of TNBC have not been clarified. We found that down-regulation of ZNF662 in breast cancer was associated with abnormal promoter methylation. ZNF662 over-expression inhibited triple-negative breast cancer cell proliferation, migration and invasion and induced cell cycle arrest in vitro, and also suppressed the growth and metastasis of xenograft tumors in vivo. Further experiments confirmed that ZNF662 could directly bind to the NGF promoter sites and significantly inhibit NGF transcription activity. In addition, ZNF662 could increase the sensitivity of TNBC cells to the EGFR inhibitor lapatinib. Molecular mechanisms revealed that ZNF662 affected downstream PI3K/AKT and STAT3 signaling pathways to inhibit TNBC progression by down-regulating NGF expression. Altogether, we speculated that ZNF662 might become a promising prognostic marker and therapeutic target for early detection and treatment of TNBC.

## Introduction

Breast cancer is the most frequent malignant tumor among women worldwide. In 2022, it was estimated that more than 2.3 million new cases occurred 1. Although advances in surgical and non-surgical treatment have greatly improved the prognosis of patients, there are still over 665,000 deaths annually 1-3. Among different subtypes of breast cancer, triple-negative breast cancer (TNBC) is more intractable. TNBC is regarded as the most aggressive subtype, exhibiting increased recurrence and decreased survival. There has been no significant progress in the treatment of TNBC patients during recent years, mainly due to the lack of effective targeted therapy 4, 5. Notably, over 70% of TNBC tumors exhibit EGFR over-expression, which correlates with poor prognosis, suggesting its potential utility in both diagnosis and targeted therapy 6, 7. These findings have spurred numerous investigations into EGFR-targeted therapies for TNBC, including lapatinib-a dual inhibitor of HER2 and EGFR 8, 9. The specificity of EGFR targeting and observed clinical benefits in cancer progression and metastasis highlight lapatinib as a promising therapeutic candidate 10-12. Regrettably, the clinical application of lapatinib in TNBC is still limited due to drug resistance 10. Therefore, identifying effective therapeutic targets to increase the sensitivity of TNBC to lapatinib is critical for optimizing TNBC management.

Tumor epigenetics are regulatory mechanisms of tumorigenesis and development that alter gene expression levels without changes in underlying DNA sequence 13. DNA methylation, as a key component of tumor epigenetics, is increasingly recognized for its influential role in malignant tumors. It is well known that DNA hypomethylation may lead to activate oncogenes, while DNA hyper-methylation could silence tumor suppressor genes 14, 15. Zinc Finger Proteins (ZFPs) are the largest family of transcription factors in the human genome, one-third of which are KRAB-ZFPs. Previous studies have reported that KRAB-ZFPs participate in regulating diverse biological events, such as cell development and differentiation 16, 17. Considering the important role of KRAB-ZFPs family members in transcription regulation, increasing researches have been done to explore the relationship between aberrant DNA methylation of KRAB-ZFPs family and cancer progression. For instance, ZNF471 activates MAPK10/JNK3 signaling pathway but is frequently silenced by promoter CpG methylation in esophageal cancer 18. The transcription inhibitor ZNF382 is also down-regulated in multiple tumors due to promoter methylation 19. Moreover, it is worth mentioning that dynamic and reversible DNA methylation status provides potential targets for cancer therapy 20. Thus, studying the mechanism by which KRAB-ZFPs contribute to cancer development is important for better understanding cancer pathogenesis and finding effective targets for early diagnosis and therapy.

From this perspective, we analyzed our own DNA methylation sequencing data of TNBC and performed combined analysis with TCGA database. Accordingly, we identified multiple candidate genes that may serve as potential therapeutic targets. These genes are characterized by DNA hyper-methylation, low expression, and poor prognosis in TNBC. In the present study, we identified a novel tumor suppressor gene of the KRAB-ZFPs family in TNBC, ZNF662. ZNF662 is located on the chromosomal region 3p22.1. Previous researches reported that deletions or mutations in this region were associated with tumor initiation and development in renal cell carcinoma 21, multiple myeloma 22 and non-small cell lung cancer 23. In addition, ZNF662 has been reported as a valuable biomarker in oral squamous cell carcinoma 24-26. Furthermore, ZNF662 was recognized in the meta-analysis of ChIP-seq data of ER+ cell lines stimulated with E2 27. However, its biological function and potential mechanism in TNBC have not been clarified.

In this research, we elucidated the anti-carcinogenic effect of ZNF662 on TNBC *in vivo* and* in vitro*. Our results may provide novel insights into the treatment of TNBC.

## Materials and Methods

### Cell lines, tumor samples

Seven breast cancer cell lines (BT549, MB231, MCF7, T-47D, ZR-75-1, MB468, SK-BR-3) and normal cell lines (MCF10A and HMEC) were acquired from American Type Culture Collection (ATCC). YCCB1 cell lines were kindly provided by Prof. Qian Tao (The Chinese University of Hong Kong). All breast cancer cell lines were cultured in RPMI-1640 (Gibco-BRL, MD, USA) or DMEM (Gibco-BRL) medium with 10% fetal bovine serum (Gibco-BRL), 1% penicillin and streptomycin (Gibco-BRL) at 37 °C in a humidified atmosphere containing 5% CO_2_. MCF10A and HMEC were cultured in human mammary epithelial growth medium (Lonza, Basel, Switzerland). Cell lines were authenticated by Short Tandem Repeat (STR) profiling and confirmed negative for mycoplasma. All clinical samples, including primary tumor tissues and paired adjacent noncancerous tissues, were acquired from the First Affiliated Hospital of Chongqing Medical University and stored at -80 °C until evaluation by pathologists. These tissues were histologically examined and pathologically collected. All enrolled patients accepted written informed consent.

### RNA extraction

Total RNA was extracted from cell lines with TRIzol reagent (Invitrogen, Carlsbad, CA, USA) following the manufacturer's instructions. Samples were stored at -80 °C until further use.

### Reverse transcription (RT) and quantitative real-time PCR (qRT-PCR)

cDNA (20 μL) was synthesized from 1 μg RNA with the Reverse Transcription System (Promega, Madison, WI, USA). qRT-PCR was performed on ABI 7500 Real-Time PCR system (Applied Biosystems) using SYBR Green (Promega) according to the manufacturer's instructions 28. The primer sequences are shown in Additional file 1: Table S1.

### Methylation-specific PCR (MSP)

MSP was performed to evaluate ZNF662 methylation status as described previously 28. Genomic DNA was extracted from tissues using the QIAamp DNA Mini Kit (Qiagen, Hilden, Germany). Bisulfite-treated DNA was amplified by MSP using the methylation-specific primers shown in Additional file 1: Table S1. The MSP was conducted using AmpliTaq Gold DNA Polymerase (Applied Biosystems). PCR products were electrophoresed on 2% agarose gel and visualized using Gel Doc XR Imaging System (Bio-Rad, CA, USA).

### 5-Aza-2*'*-deoxycytidine (Aza) and trichostatin A (TSA) treatment

MB231 and YCCB1 cell lines were treated with 10 μM Aza (DNA methyltransferase inhibitor, Sigma-Aldrich, Steinheim, Germany) for 72 h. Then, the cells were further treated with 100 nM TSA (histone deacetylase inhibitor, Sigma-Aldrich) for 24 h. RNA extraction was performed for further analysis.

### Lentivirus and cell lines stably expressing ZNF662

293T cells were used for lentivirus packaging. The pEZ-Lv242-ZNF662-Flag or vector plasmids were co-transfected with LipoD293™ (SignaGen Laboratories, Rockville, MD, USA) into 293T cells along with psPAX2 and pMD2.G packing plasmids. Cell infection and screening were performed as previously described 29. All antibiotic-resistant cells were verified by both qRT-PCR and Western blot.

### Construction of plasmids, transfection and recombinant protein

The NGF gene was inserted into the pcDNA3.1 plasmid. The recombinant plasmids were transformed into E. coli DH5α competent cells. NGF expression plasmids or control plasmids were transfected into MB231 and YCCB1 cells with Lipofectamine 2000 (Invitrogen). Recombinant human NGF protein was purchased from Abclonal (RP01792, Wuhan, China) and used at a concentration of 100 ng/ml.

### SiRNA

ZNF662 siRNA (siZNF662) was obtained from OriGene Technologies (Rockville, MD, USA). siRNA transfection was carried out with Lipofectamine 2000 (Invitrogen). Cells were harvested at 48-72 h post-transfection for future experiments.

### Cell viability assays

Counted cells were cultured in 96-well plates at 2 × 10^3^ cells/well. The absorbance in each well was measured at 450 nm using Cell Counting Kit-8 (CCK-8; Dojindo Laboratories, Kumamoto, Japan) at 0 h, 24 h, 48 h, and 72 h. Experiments were performed in triplicate.

### Chemosensitivity assay

To test the influence of ZNF662 on the sensitivity of TNBC cells to lapatinib, 4 × 10^3^ MB231 and YCCB1 cells per well were planted in 96-well plates. After adhering overnight, cells were treated with different concentrations of lapatinib (MCE, Shanghai, China). Cell viability was measured by CCK-8 assay at 24 h following the manufacturer's instructions. Then, the inhibition rate was calculated based on the absorbance at 450 nm.

### Colony formation assays

Cells were seeded in 6-well plates at 4 × 10^2^ cells/well. After 2 weeks, cells were washed two times with phosphate-buffered saline (PBS) and fixed with 4% paraformaldehyde (PFA) for 30 min. Then, cells were stained with crystal violet for 30 min. Colonies were counted and quantified using ImageJ software (NIH, MD, USA).

### Transwell^®^ migration and invasion assay

Matrigel-uncoated and -coated transwell chambers (8 μm, BD Sciences, Bedford, MA, USA) were conducted to evaluate cell migration and invasion ability. To estimate migration efficiency, cells were added into the upper wells. To estimate invasion efficiency, cells were seeded in the pre-coated upper compartment. Lower chambers were filled with 700 μL RPMI-1640 medium supplemented with 20% fetal bovine serum (FBS). After 48 h of incubation, cells were fixed in 4% paraformaldehyde for 30 min and stained with crystal violet for 30 min. Migrated cells were counted and quantified using ImageJ software (NIH).

### Flow cytometry analysis

Cells were collected after being digested with trypsin, suspended in ice-cold 70% ethanol, and fixed for 24 h. Then, cells were treated with RNase A (Sigma-Aldrich), incubated at 37 °C for 30 min, and stained with propidium iodide (PI) for 30 min in the dark. Cell cycle distribution was analyzed by flow cytometry (FCM).

### Tumor xenograft model in nude mice

Five female BALB/c nude mice (4-6 weeks of age, weighing 18-22 g) were used to study whether ZNF662 inhibits tumor growth *in vivo*. All animal experiments were approved by the Institutional Ethics Committees of Chongqing University Cancer Hospital. Stable ZNF662-expressing MB231 cells and control cells (2 ×10^6^ cells re-suspended in 0.1 mL PBS) were injected subcutaneously into the back on both sides of five nude mice. Tumor measurements were performed every third day, starting at day three after injection. Tumor volume was calculated as following formula: volume = longest diameter × shortest diameter^2^ × 0.52. All nude mice were sacrificed on day 20. The subcutaneous tumors were measured. Samples were frozen at -80 °C for subsequent analysis.

For metastasis model, 1 × 10^6^ ZNF662-expressing and control MB231-luc cells were injected intracardially or intravenously into nude mice. Bioluminescence imaging was performed using an IVIS Imaging System (PerkinElmer, Waltham, MA, USA). Nude mice were sacrificed at the indicated experimental endpoint. The collected lung tissues were examined histologically by H&E staining.

### Immunohistochemistry (IHC)

Immunohistochemical staining was performed using a streptavidin-peroxidase kit (Maixin-Bio, Fujian, China). Sections were dewaxed, rehydrated, underwent antigen retrieval, blocked, and incubated with primary antibody at 4 °C overnight, then incubated with secondary antibody at 37 °C for 30 min. The section was counterstained with hematoxylin. Anti-Flag was acquired from Abm (#G188, Abm, Richmond, BC, Canada) and anti-Ki67 was acquired from Abcam (ab15580, Abcam, Cambridge, UK).

### Western blot

Cells were washed three times with PBS and then lysed in RIPA Lysis Buffer (Beyotime, Shanghai, China) containing protease inhibitors (phenyl methane sulfonyl fluoride, cocktail). Proteins were separated using SDS-PAGE electrophoresis (Bio-Rad) and then transferred onto PVDF membranes (Millipore, MA, USA). Western blot assays were performed as described previously 30. Primary antibodies used are as follows: STAT3 (#9139, Cell Signaling Technology), Phospho-STAT3 (#9145, Cell Signaling Technology), EGFR (#4267, Cell Signaling Technology), Phospho-EGF Receptor (#3777, Cell Signaling Technology), AKT (#4691, Cell Signaling Technology), Phospho-AKT (#4060, Cell Signaling Technology), p38 MAPK (#8690, Cell Signaling Technology), Phospho-p38 MAPK (#4511, Cell Signaling Technology), NF-κB p65 (#8242, Cell Signaling Technology), Phospho-NF-κB p65 (#3033, Cell Signaling Technology), Erk1/2 (#4695, Cell Signaling Technology), Phospho-Erk1/2 (#4370, Cell Signaling Technology), SNAIL (#3895, Cell Signaling Technology), Slug (#9585, Cell Signaling Technology), anti-Flag (#G188, Abm), anti-Flag M2 (#14793, Cell Signaling Technology), ZNF662 (PA5-58662, Thermo Fisher), TrkA (bs10210R, BIOSS), Phospho-TrkA (bs-17445R, BIOSS), p75 NGF receptor (bs-0161R, BIOSS). β-actin (#sc-8432, Santa Cruz Biotechnology) and GAPDH (#sc-47724, Santa Cruz Biotechnology) served as an internal standard.

### Chromatin Immunoprecipitation (ChIP) assay

Chromatin immunoprecipitation (ChIP) was conducted using SimpleChIP^®^ Enzymatic Chromatin IP Kit (#9003, Cell Signaling Technology) according to the manufacturer's instructions. First, stable ZNF662-expressing MB231 cells were washed with ice-cold PBS lacking Ca^2+^ /Mg^2+^ and supplemented with protease inhibitor cocktail (Sigma-Aldrich). Then, protein-DNA complexes were cross-linked using 1% formaldehyde for 10 min at room temperature, and 0.125 M glycine was used to terminate the reaction. Cells were resuspended in lysis buffer for 10 min, and incubated with micrococcal nuclease for 20 min at 37 °C. In order to obtain 200-900 bp DNA fragments, cells were disrupted by sonication on ice using an ultrasonic cell disruptor (6 pulses for 10 sec, 40% amplitude). 10 μL of the sample was removed in advance to serve as the input control. The sample was incubated with anti-Flag M2 (#14793, Cell Signaling Technology) overnight at 4 °C. Protein A/G magnetic beads (#9006, Cell Signaling Technology) were added to each sample, followed by incubation for 2 h at 4 °C. Positive control was incubated with rabbit anti-histone 3 H3 antibody (#4620, Cell Signaling Technology), and negative control was incubated with normal rabbit IgG (#2729, Cell Signaling Technology). Precipitated complexes were washed extensively and eluted from the beads as recommended. The crosslinking of the protein-DNA complexes was reversed overnight at 65 °C, followed by treatment with 6 μL of 5 M NaCl and 2 μL of proteinase K. DNA was then purified for subsequent qRT-PCR analysis. Primer sequences are listed in Additional file 1: Table S1.

### Luciferase reporter assay

Human NGF luciferase reporter plasmid was constructed essentially. Cells were seeded in 24-well plates and transfected with luciferase reporter plasmids. Renilla luciferase plasmid served as an internal control. Cells were lysed 48 h after transfection with 100 μL 1 × Passive Lysis Buffer (Promega). Then, luciferase activity was measured using Dual-Luciferase Reporter Assay Kit (Promega). The light emission was determined by a spectrophotometer (Infinite M200 PRO, Tecan, Austria).

### RNA-sequence analysis

RNA-Seq was performed by BGI company (Shenzhen, China). The RNA integrity number was assessed by Agilent Bioanalyzer 2100 system (Agilent Technologies, Santa Clara, CA, USA) with an RNA Nano 6000 Assay Kit. RNA sample was used to construct the cDNA library. The quality of the library was assessed by Agilent Bioanalyzer 2100 system. The library was sequenced using the BGISEQ-500 platform (BGI), and single-end reads were generated. Genes with an adjusted *p-*value* < 0.05* found by DESeq were assigned as differentially expressed.

### Pathway enrichment analysis

Kyoto Encyclopedia of Genes and Genomes (KEGG) pathways 31, which were obtained from DAVID database 32, were used to analyze the functional enrichment of GO terms among differentially expressed genes 33. The significance level was *p < 0.05*.

### Statistical analyses

GraphPad Prism 8 was used to analyze the statistics. Data were presented as mean ± standard error of the mean (SEM). Statistical analysis was performed by comparing the experimental and control values using Student's t-test. The significance of statistical analysis was clarified by *p < 0.05, **p < 0.01 and ***p < 0.001.

## Results

### The expression of ZNF662 is down-regulated in breast cancer tissues and cells

**Figure 1:**
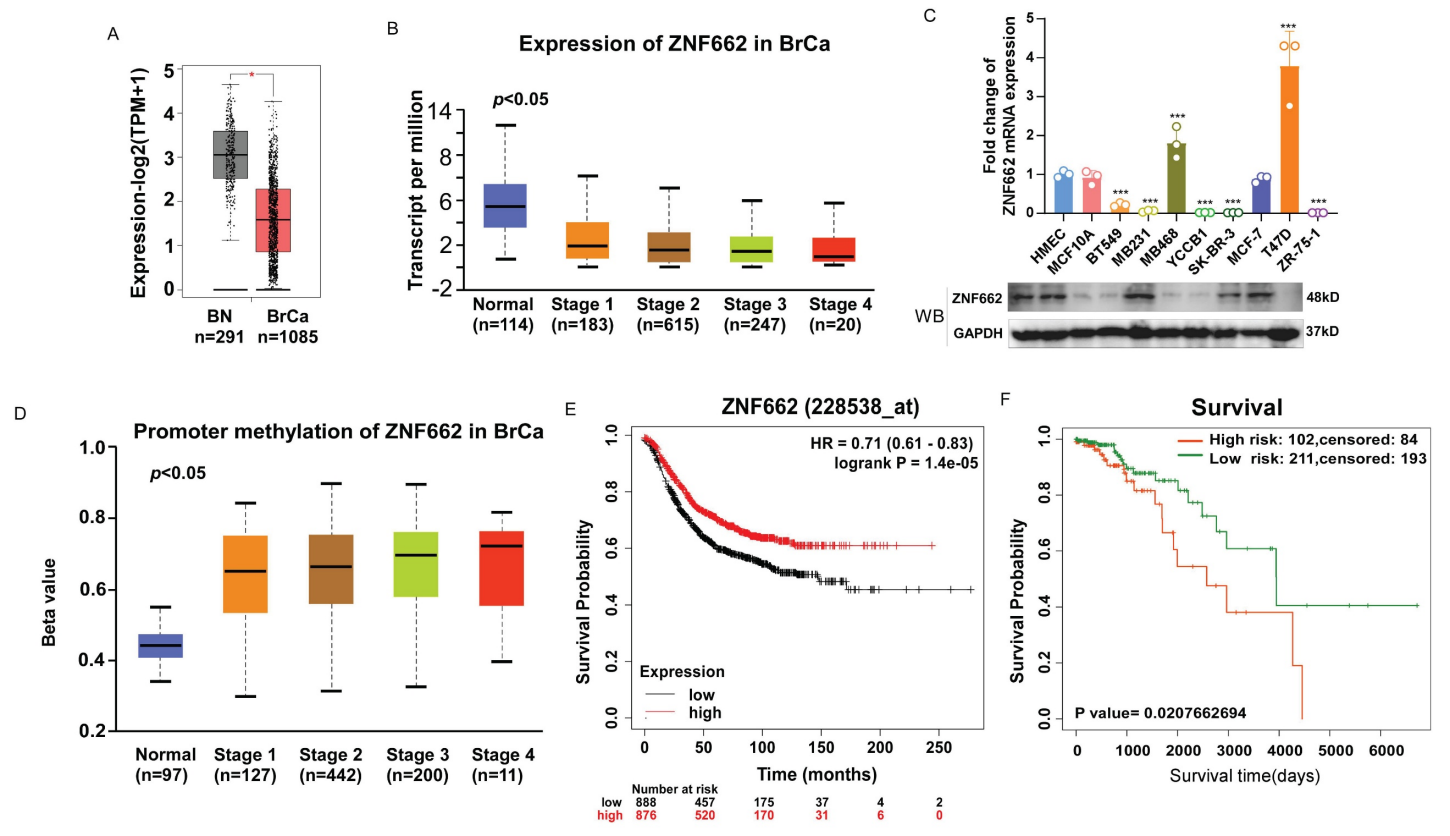
** The expression and promoter methylation status of ZNF662 in breast cancer tissues and cell lines.** (A) The expression difference of ZNF662 in 1085 BRCA tissues and 291 normal breast tissues from GEPIA2 database. (B) ZNF662 expression stratified by normal breast tissues and the different stages of BRCA tissues from UALCAN database. (C) ZNF662 expression in breast cancer cell lines (n = 8) and normal breast epithelial cells (n = 2) was detected by qRT-PCR and Western blot. The ZNF662 mRNA expression level was determined relative to that in HMEC cells. (D) ZNF662 promoter methylation status stratified by normal breast tissues and the different stages of BRCA tissues from UALCAN database. (E) The higher ZNF662 expression was associated with a better prognosis in breast cancer patients from Kaplan-Meier Plotter database (http://kmplot.com/analysis/). The median expression of ZNF662 was selected as the threshold to split the high-expression and low-expression cohorts. (F) The higher promoter methylation status of ZNF662 was associated with a worse prognosis in breast cancer patients from SurvivalMeth database (http://bio-bigdata.hrbmu.edu.cn/survivalmeth/). A total of 313 BRCA patients were divided into high- and low-risk groups based on the optimal cutoff (prognostic index) determined by SurvivalMeth database. BRCA, breast invasive carcinoma; BN, normal breast tissue; HR, hazard ratio. Error bars represent standard deviation (SD); data are presented as the mean ± SD. ***p < 0.001.

To investigate the role of ZNF662 in breast cancer, we first evaluated the expression of ZNF662 in breast cancer tissues using GEPIA2 database (http://gepia2.cancer-pku.cn/). It was illustrated that ZNF662 was lowly expressed in breast cancer tissues compared to normal breast tissues. Moreover, we observed that the expression of ZNF662 was negatively correlated with the tumor stage using UALCAN database (http://ualcan.path.uab.edu/analysis.html) (Fig. 1A+B). Additionally, we detected the expression of ZNF662 in breast cancer cell lines. We found that the expression of ZNF662 was almost absent in most breast cancer cell lines (Fig. 1C). As mentioned in the Introduction, ZNF662 was identified from DNA methylation sequencing data and promoter hyper-methylation was associated with gene silencing. Therefore, we explored the methylation status of ZNF662. Online analysis demonstrated promoter methylation status of ZNF662 was positively correlated with tumor progression (Fig. 1D). Next, we assessed the prognostic significance of ZNF662 expression and methylation level in patients with breast cancer. The survival analysis indicated breast cancer patients with high expression of ZNF662 had a better survival probability (Fig. 1E), while hyper-methylation of ZNF662 promoter might contribute to a worse prognosis (Fig. 1F).

### Promoter hyper-methylation results in the reduced expression of ZNF662 in breast cancer

**Figure 2:**
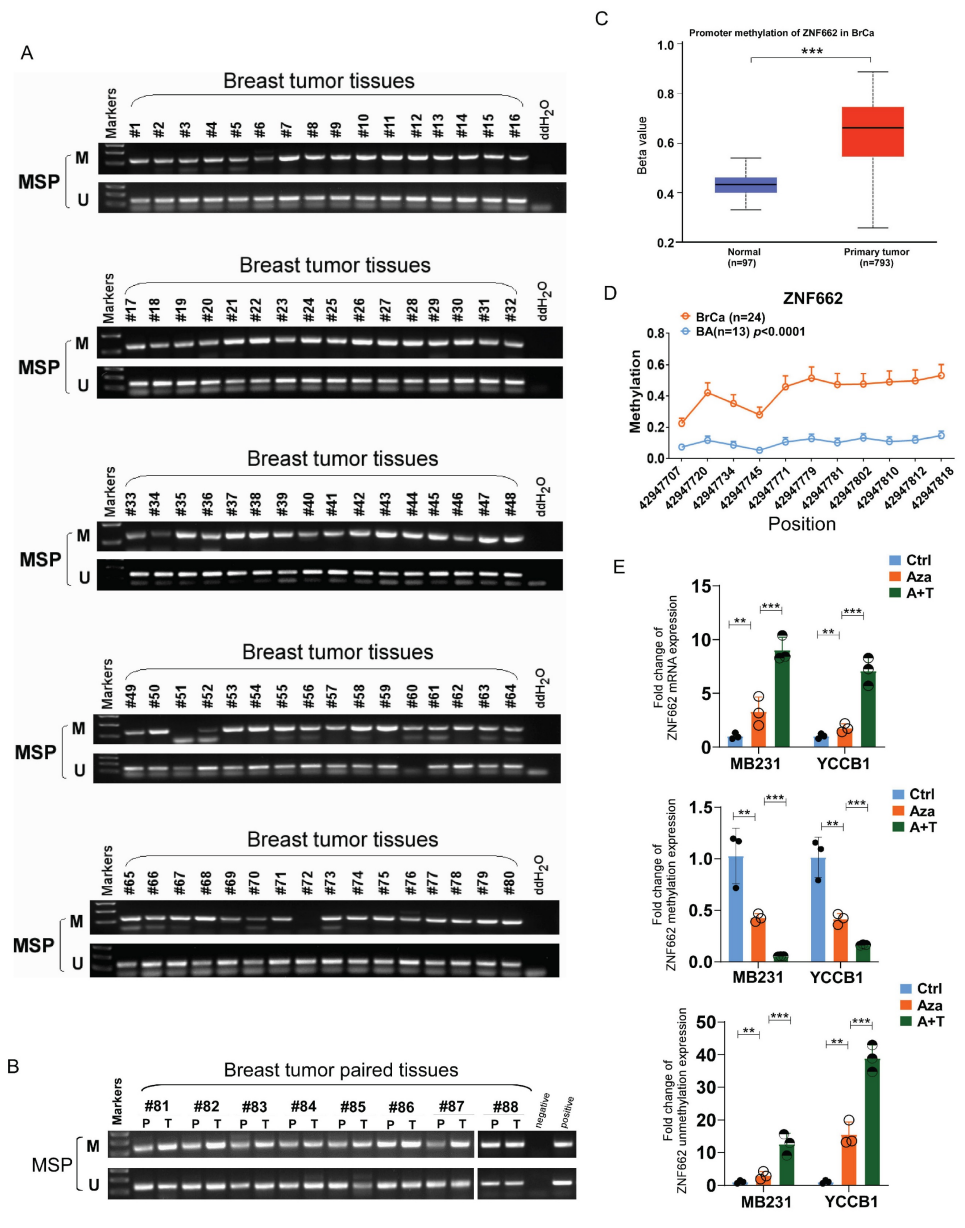
** Promoter hyper-methylation contributed to reduced expression of ZNF662 in breast cancer.** (A+B) Promoter methylation status of ZNF662 in breast tumor tissues (n = 80) and paired breast tumor tissues (n = 8) was detected by MSP assay. (C) Promoter methylation status of ZNF662 was analyzed using UALCAN database. (D) Methylation status at multiple sites of ZNF662 using MethylTarget^®^ analysis. (E) ZNF662 mRNA, methylation and unmethylation expression in MB231 and YCCB1 cells after Aza (A) and/or TSA (T) treatment was detected by qRT-PCR. Aza, 5-aza-2*'*-deoxycytidine; TSA, trichostatin A; BRCA, breast invasive carcinoma; BA, breast normal tissue. Error bars represent standard deviation (SD); data are presented as the mean ± SD. *p < 0.05, **p < 0.01, ***p < 0.001.

To further demonstrate the association between ZNF662 expression and its promoter methylation status, we performed Methylation‑specific PCR (MSP) assay. Results showed that ZNF662 promoter methylation status was detected at a high level in breast tumor tissues, and compared with paired adjacent breast tissues, ZNF662 promoter methylation status was significantly higher in breast cancer tissues (Fig. 2A+B). Similar conclusion was drawn from online analysis on UALCAN database (Fig. 2C). Furthermore, we found significantly higher methylation levels at multiple sites of ZNF662 in breast cancer tissues using MethylTarget^®^ analysis (Fig. 2D). We also detected methylated CpG sites of ZNF662 in BRCA tissues using MethSurv database (https://biit.cs.ut.ee/methsurv/) (Additional file 2: sFig. 1B). Further survival analysis revealed that the methylation level of CpG sites was correlated with prognosis in BRCA patients (Additional file 2: sFig. 1C). Down-regulation of tumor suppressor genes due to promoter hyper-methylation in malignant tumors is one of the most common and important mechanisms in cancer occurrence and development. To further determine whether ZNF662 silencing was a result of promoter methylation, we treated breast cancer cells with DNA methyltransferase inhibitor Aza and histone deacetylase inhibitor TSA. Results showed that the expression of ZNF662 in MB231 and YCCB1 cells could be restored after treatment with drugs, accompanied by decreased methylation and increased unmethylation (Fig. 2E). This might be one of the reasons for the reversal of malignant biological behaviors (Additional file 2: sFig. 2). Moreover, cBioPortal database (https://www.cbioportal.org/) indicated that ZNF662 methylation status was negatively correlated with ZNF662 expression in BRCA tissues (Additional file 2: sFig. 1A). In summary, these results indicated that ZNF662 was down-regulated through promoter hyper-methylation in breast cancer, suggesting that ZNF662 might function as a tumor suppressor gene.

### Over-expression of ZNF662 could suppress the proliferation of TNBC cells and induce G0/G1 or G2/M cell cycle arrest

**Figure 3:**
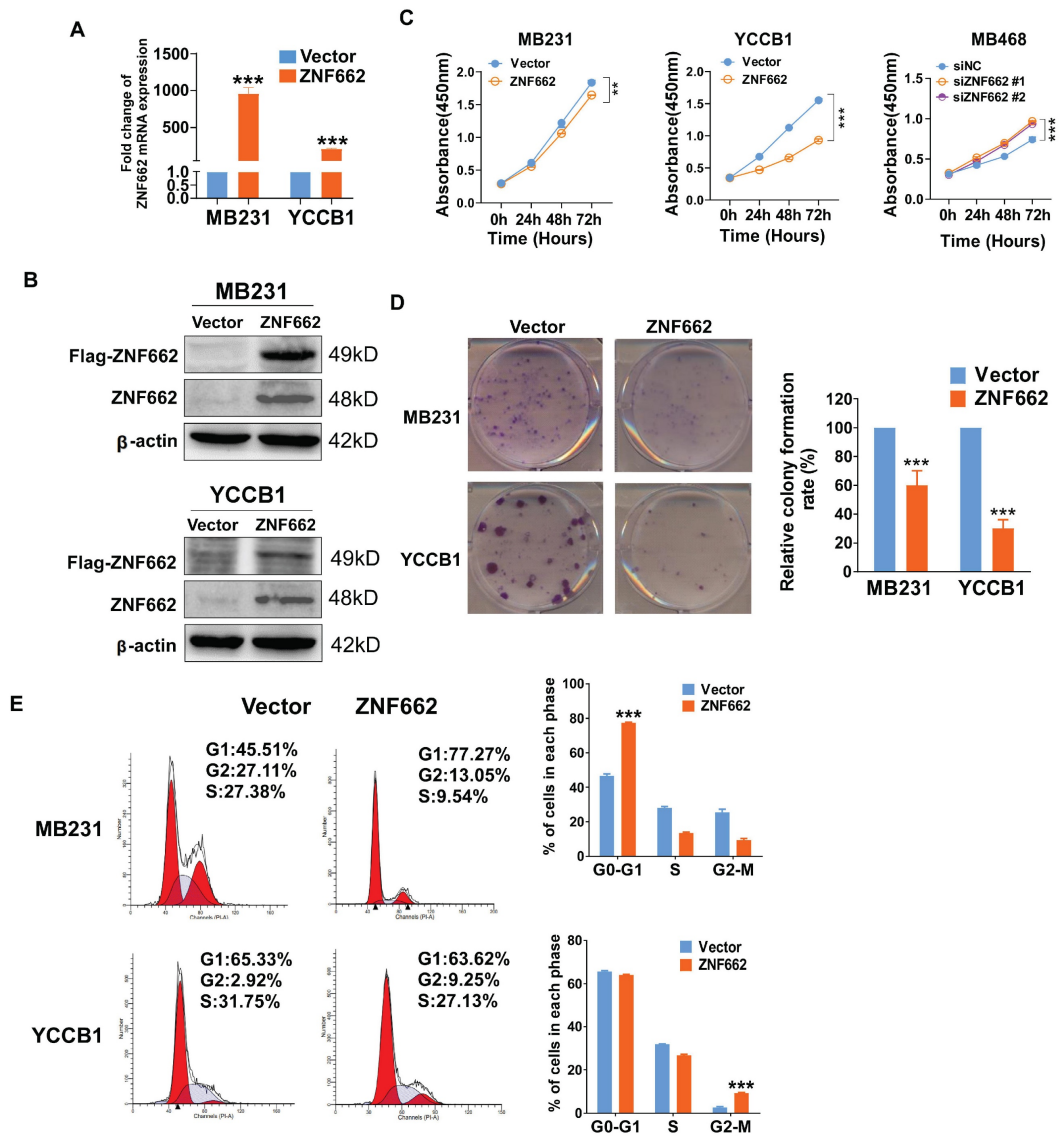
** ZNF662 inhibits TNBC cell proliferation and induces G0/G1 or G2/M cell cycle arrest.** (A+B) Validation of ZNF662 over-expression by qRT-PCR and Western blot. (C) CCK-8 assay revealed the effects of ZNF662 over-expression and knockdown on the proliferation of TNBC cells. (D) Colony formation assay of vector and ZNF662-overexpressing MB231 and YCCB1 cells. Left: representative images. Right: histogram statistics. (E) Cell cycle distribution of vector and ZNF662-overexpressing MB231 and YCCB1 cells. Left: representative flow cytometry plots; right: histogram statistics. All data are shown as mean ± SD from three independent experiments. SD, standard deviation. *p < 0.05, **p < 0.01, ***p < 0.001.

To explore the function of ZNF662 in TNBC, we established MB231 (ER-/PR-/HER2-) and YCCB1 (ER-/PR-/HER2-) cell lines stably expressing ZNF662, which were verified by qRT-PCR and Western blot (Fig. 3A+B). Similarly, siZNF662 was transfected into MB468 (ER-/PR-/HER2-) cell lines, and the transfection efficiency was confirmed (Additional file 2: sFig. 3A+B). In terms of cell proliferation and viability, CCK-8 and colony formation assays showed that ZNF662 over-expression could hinder the growth of TNBC cells (Fig. 3C+D). However, ZNF662 knockdown could promote the growth of MB468 cell lines (Fig. 3C, sFig. 3C). We further detected cell cycle distribution by flow cytometry. Results showed that ZNF662 over-expression resulted in elevated cell numbers in G0/G1 and G2/M phase (Fig. 3E). In contrast, ZNF662 knockdown resulted in elevated cell numbers in S phase (Additional file 2: sFig. 3D). These results indicated that ZNF662 exerted a tumor-suppressive effect by inhibiting cell proliferation and inducing cell cycle arrest in TNBC cells.

### ZNF662 could impair the metastatic ability of TNBC cells *in vitro*

**Figure 4:**
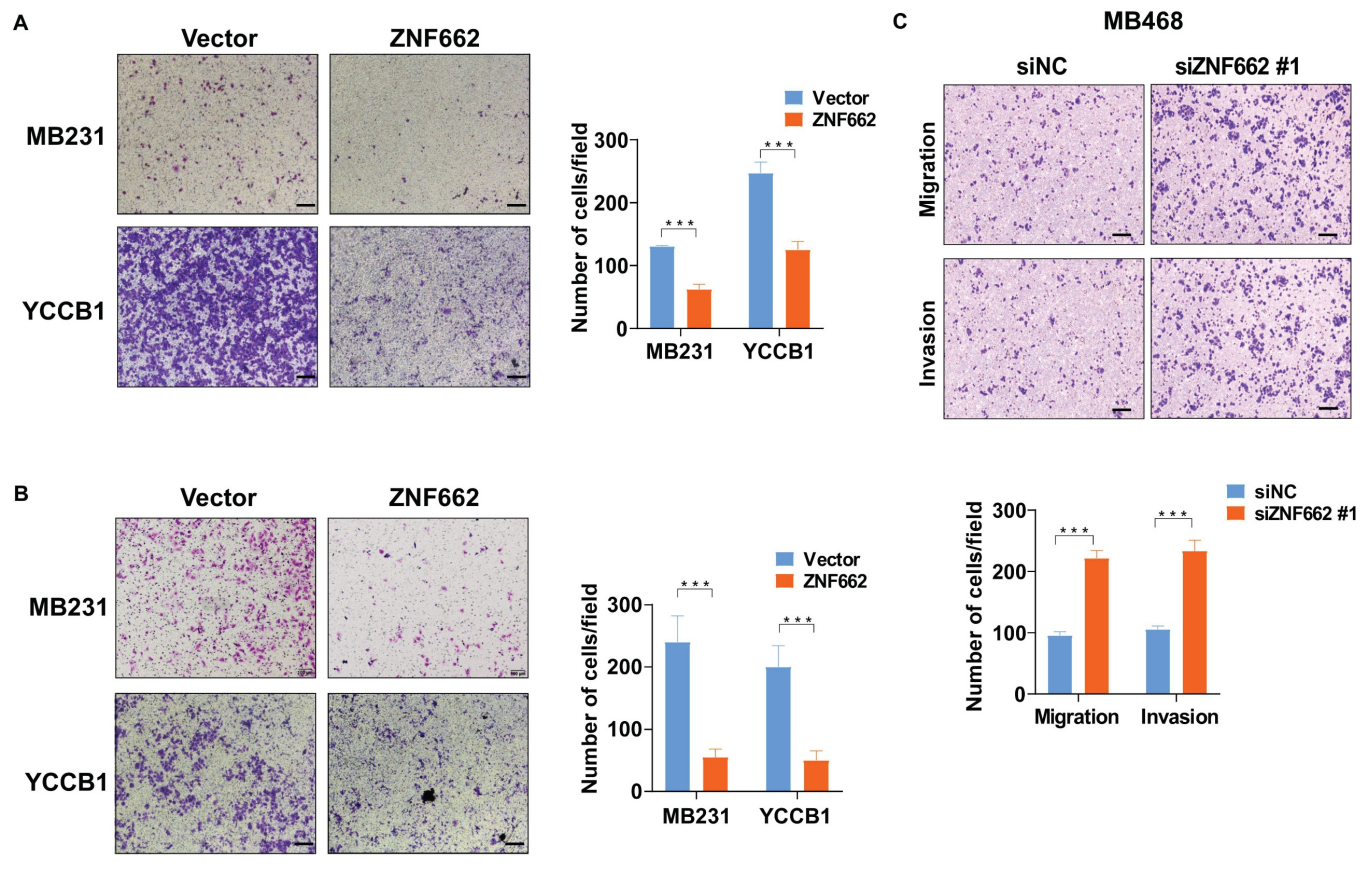
** ZNF662 suppresses the migration and invasion of TNBC cells *in vitro*.** (A+B) Representative image (left) and histogram statistics (right) of Transwell assay in vector and ZNF662-overexpressing MB231 and YCCB1 cells. The top panels represent Transwell migration assay, and the bottom panels represent Transwell invasion assay. (C) Representative image (above) and histogram statistics (below) of Transwell assay in control and ZNF662 knockdown cells. Scale bars: 100 μm. All data are presented as the mean ± SD from three independent experiments. SD, standard deviation. ***p < 0.001.

Next, we further pay attention to the potential impact of ZNF662 on metastasis *in vitro*. Transwell assays were used to evaluate cell migration and invasion capabilities. Results showed that ZNF662 over-expression reduced the migration and invasion of TNBC cells (Fig. 4A+B). In contrast, ZNF662 knockdown promoted the metastatic ability of MB468 cells *in vitro* (Fig. 4C).

### ZNF662 could suppress the growth and metastasis of xenograft tumors *in vivo*

**Figure 5:**
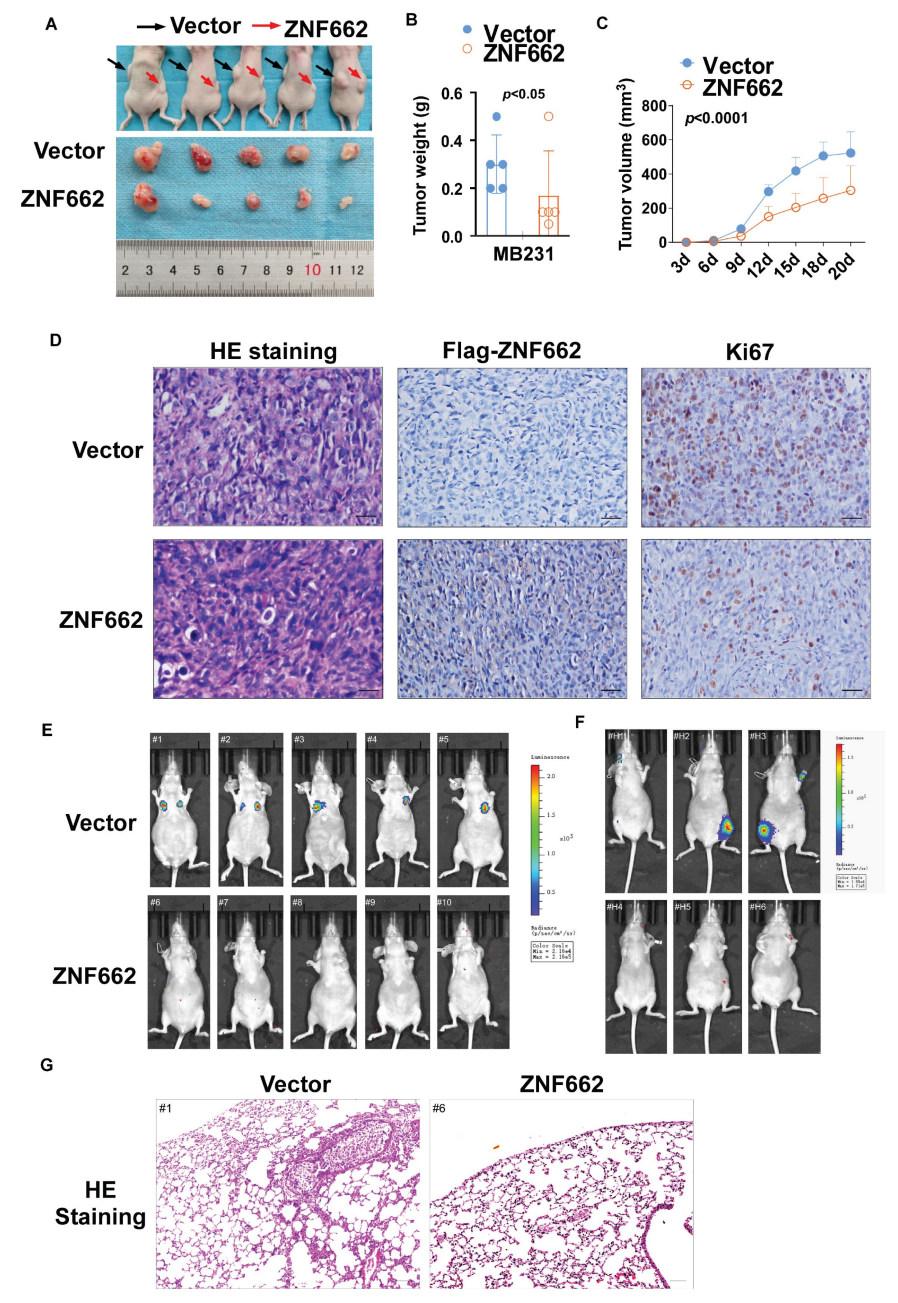
** ZNF662 inhibits the growth and metastasis of xenograft tumors in nude mice.** (A) Images of human breast tumor xenografts (n = 5). Black and red arrows indicate vector and ZNF662-overexpressing tumors, respectively. (B) Tumor weights of vector and ZNF662-overexpressing tumors. (C) Tumor growth curve of vector and ZNF662-overexpressing tumors. Tumor volume was calculated from tumor length and width. (D) Representative images of H&E and IHC staining of xenografts. (E) Representative images of lung metastasis model in nude mice (n = 5). At 8 weeks after tail vein injection, nude mice were sacrificed. (F) Representative images of whole-body metastasis model in nude mice (n = 3). At 4 weeks after intracardial injection, nude mice were sacrificed. (G) Representative images of H&E staining of lung metastasis. Scale bars: 100 μm. *p*-value was assessed by Student's t-test. All data are presented as the mean ± SD. SD, standard deviation.

Having confirmed the tumor suppressor function of ZNF662 *in vitro*, we proceeded to investigate its function *in vivo* by employing nude mouse xenograft models. The tumor growth curve and tumor weight in nude mice receiving subcutaneous injection of ZNF662-overexpressing MB231 cells were significantly reduced (Fig. 5A-C). Immunohistochemistry illustrated that Ki67, which is generally regarded as a marker of cell proliferation, was significantly decreased in ZNF662-overexpressing xenograft tumor tissues (Fig. 5D). Moreover, injection of ZNF662-overexpressing MB231-luc cells into the tail vein resulted in a significant reduction in lung metastases. And ZNF662 over-expression could decrease the number of whole-body metastatic tumors following intracardiac injection of MB231-luc cells (Fig. 5E+F). Representative H&E staining of metastatic lung nodules is shown in Fig. 5G. Based on *in vitro* and *in vivo* experiments, we proposed that ZNF662 could be a previously unidentified triple‑negative breast cancer suppressor gene.

### RNA sequencing analysis illustrated various genes had changed in ZNF662-overexpressing MB231 cells

**Figure 6:**
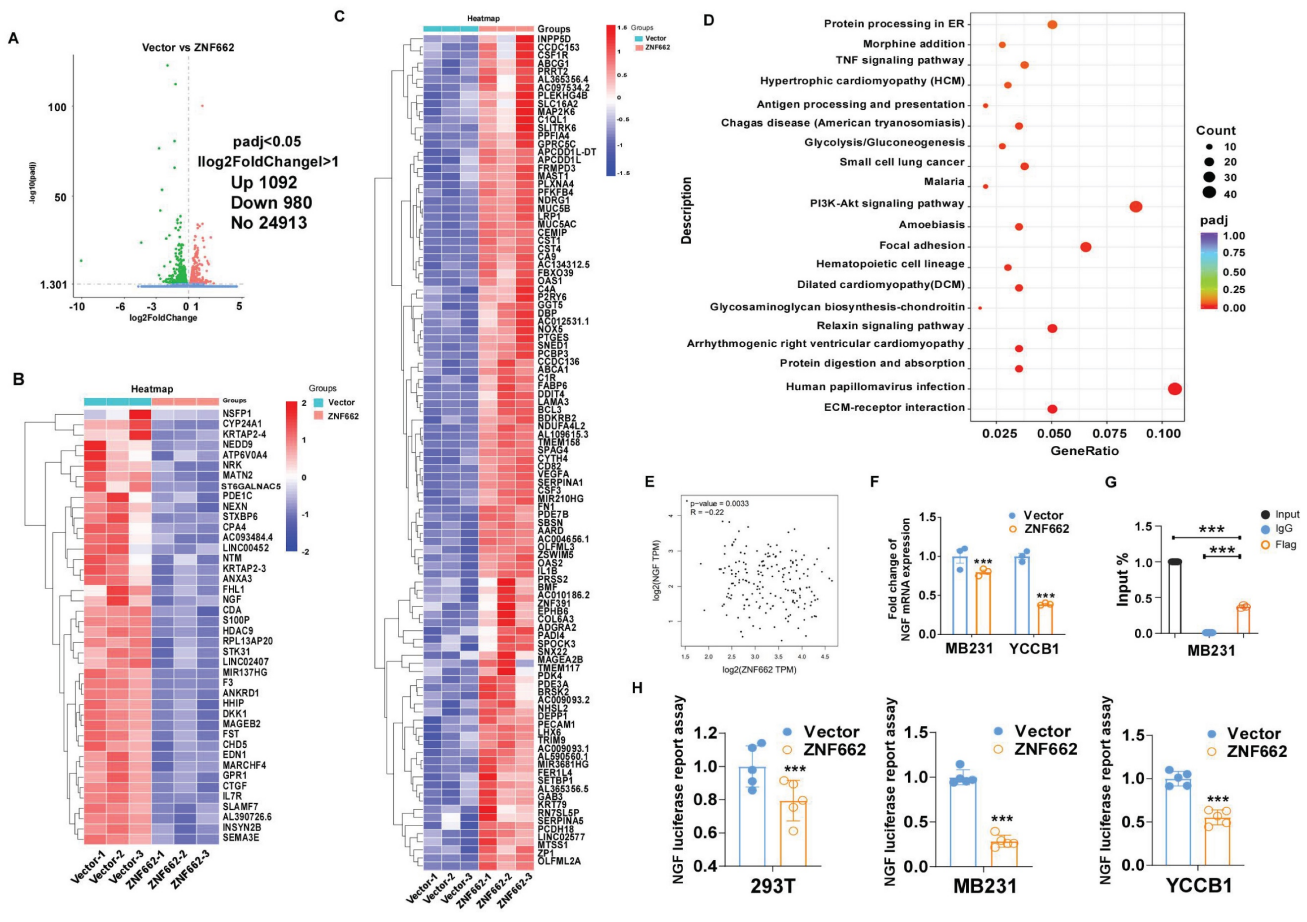
** ZNF662 could down-regulate the transcription activity of NGF in TNBC cells.** (A) Compared with control cells, the entire distribution of differentially expressed genes in ZNF662-overexpressing MB231 cells is shown as a volcano plot. (B+C) Hierarchical cluster analysis of screened differentially expressed genes in R software is shown as a heat map. (D) KEGG pathway classification of differentially expressed genes. The rich factor represents the proportion of differentially expressed genes in specific terms, and the size of the dots represents the number of relevant differentially expressed genes. (E) Pearson's correlation analysis exhibited the correlation between ZNF662 and NGF in normal mammary tissues from GEPIA2 database. (F) NGF mRNA expression in TNBC cells stably transfected with ZNF662 was detected by qRT-PCR. (G) input% of NGF DNA by anti-Flag antibody was determined by ChIP-qPCR. (H) Luciferase reporter activity assays were performed to detect the regulation of ZNF662 on NGF. Student's t-test was used. All data are presented as the mean ± SD. ***p < 0.001.

To further uncover the underlying mechanism of ZNF662 in TNBC, we used high-throughput RNA sequencing (RNA-Seq) technology to analyze the alteration of genes between control and ZNF662-overexpressing MB231 cells. Volcano plots indicated that 1092 genes were up-regulated, while 980 genes were down-regulated in the ZNF662-overexpressing MB231 cells compared with the control cells (Fig. 6A). Hierarchical clustering analysis was used to gain a comprehensive insight into the results of RNA-seq. Hierarchical clustering diagram represented a significant difference between two groups (Fig. 6B+C). The Kyoto Encyclopedia of Genes and Genomes (KEGG) pathway enrichment analysis demonstrated that the altered genes were highly enriched in the PI3K/AKT and focal adhesion signaling pathways (Fig. 6D).

### ZNF662 inhibits malignant behaviors of TNBC by suppressing NGF expression

**Figure 7:**
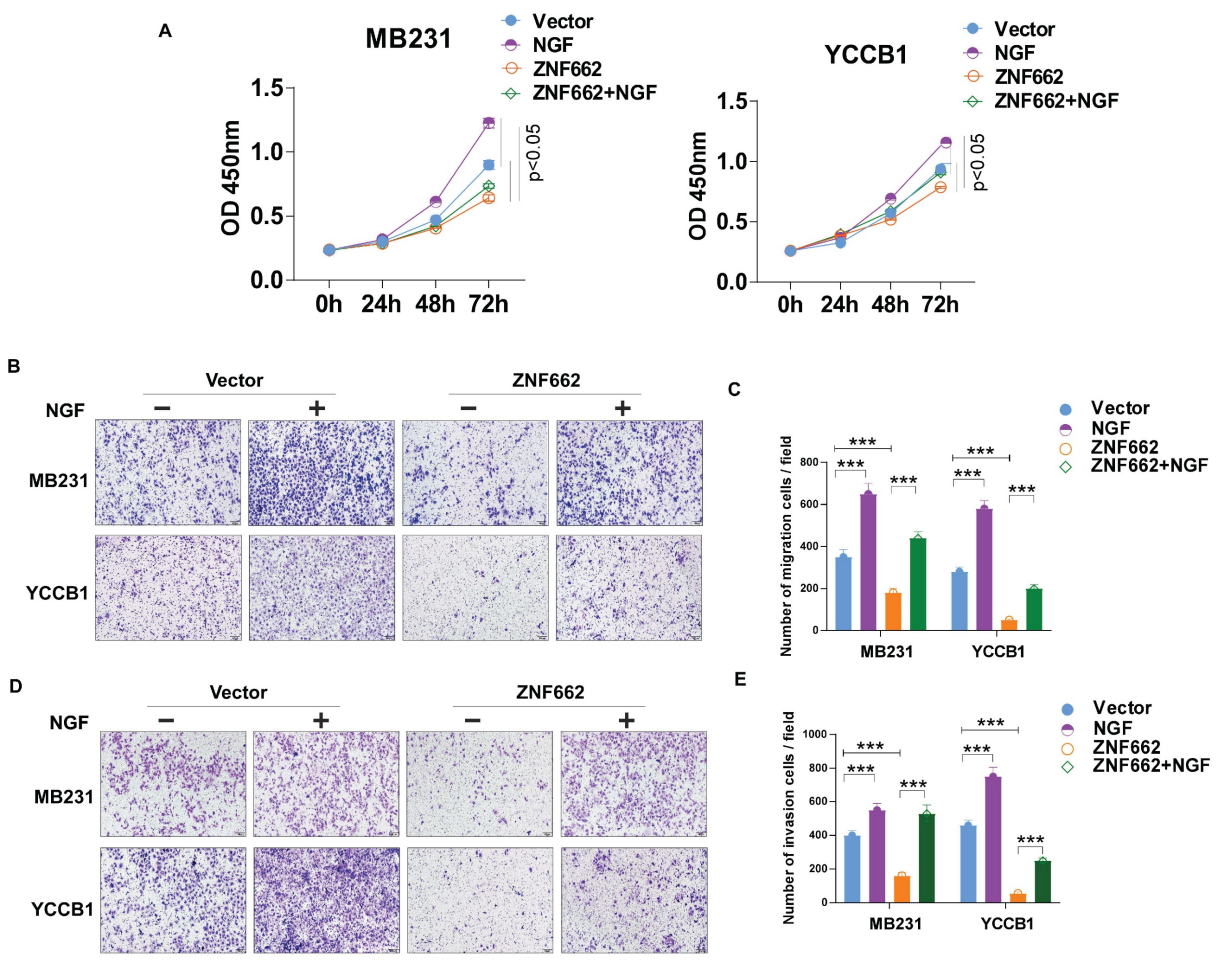
** Over-expression of NGF could reverse the tumor-suppressive effect of ZNF662 in TNBC cells.** (A) Effect of NGF over-expression on MB231 and YCCB1 cell proliferation was determined by CCK-8 assay. (B-E) Effect of NGF over-expression on MB231 and YCCB1 cell migration (B+C) and invasion (D+E) was determined by Transwell assay. *p*-value was assessed by Student's t-test. Scale bars: 100 μm. All data are presented as the mean ± SD. SD, standard deviation. *p < 0.05, **p < 0.01, ***p < 0.001.

In order to further identify potential target genes of ZNF662 in TNBC, we first ranked the RNA-seq results based on fold change and corrected p-value, then validated the top 10 differentially expressed genes (up-regulated or down-regulated) to find the same variation trend in both MB231 and YCCB1 cells by qRT-PCR (data not shown). According to qRT-PCR results, we finally selected the NGF gene as a putative target gene after a comprehensive literature search. It had been reported that NGF belonged to the family of neurotrophic factors and was considered to activate the PI3K/MAPK/PLCγ signaling pathways by binding to the TrkA receptor, thereby regulating the survival, proliferation and metastasis of breast cancer cells 34, 35. Moreover, we found a negative correlation between ZNF662 expression and NGF expression in normal mammary tissues using GEPIA2 database (Fig. 6E). Interestingly, ZNF662 expression was not related to NGF expression in BRCA tissues, which might be due to the down-regulation of ZNF662 expression. qRT-PCR showed that the ectopic expression of ZNF662 in TNBC cells resulted in down-regulation of NGF expression (Fig. 6F). It is commonly known that KRAB-ZFPs can bind to target gene promoter region to inhibit gene transcription, silence target protein expression and regulate protein post-translational modification 36. In order to further confirm that NGF is the direct inhibitory target of ZNF662, we performed dual luciferase reporter assay and ChIP-qPCR assays. Results revealed that ZNF662 could directly bind to the NGF promoter sites and significantly inhibit NGF transcription activity. (Fig. 6G+H). Further rescue assays were carried out to examine the effect of the ZNF662-NGF axis on TNBC cell proliferation and metastasis. Results suggested that over-expression of NGF could reverse the tumor-suppressive effect of ZNF662 in TNBC cells (Fig. 7A-E). In summary, our results indicated that ZNF662 may act as a tumor suppressor gene in TNBC by reducing the expression of NGF.

### The ZNF662-NGF axis regulates the PI3K/AKT and STAT3 pathways in TNBC cells

**Figure 8:**
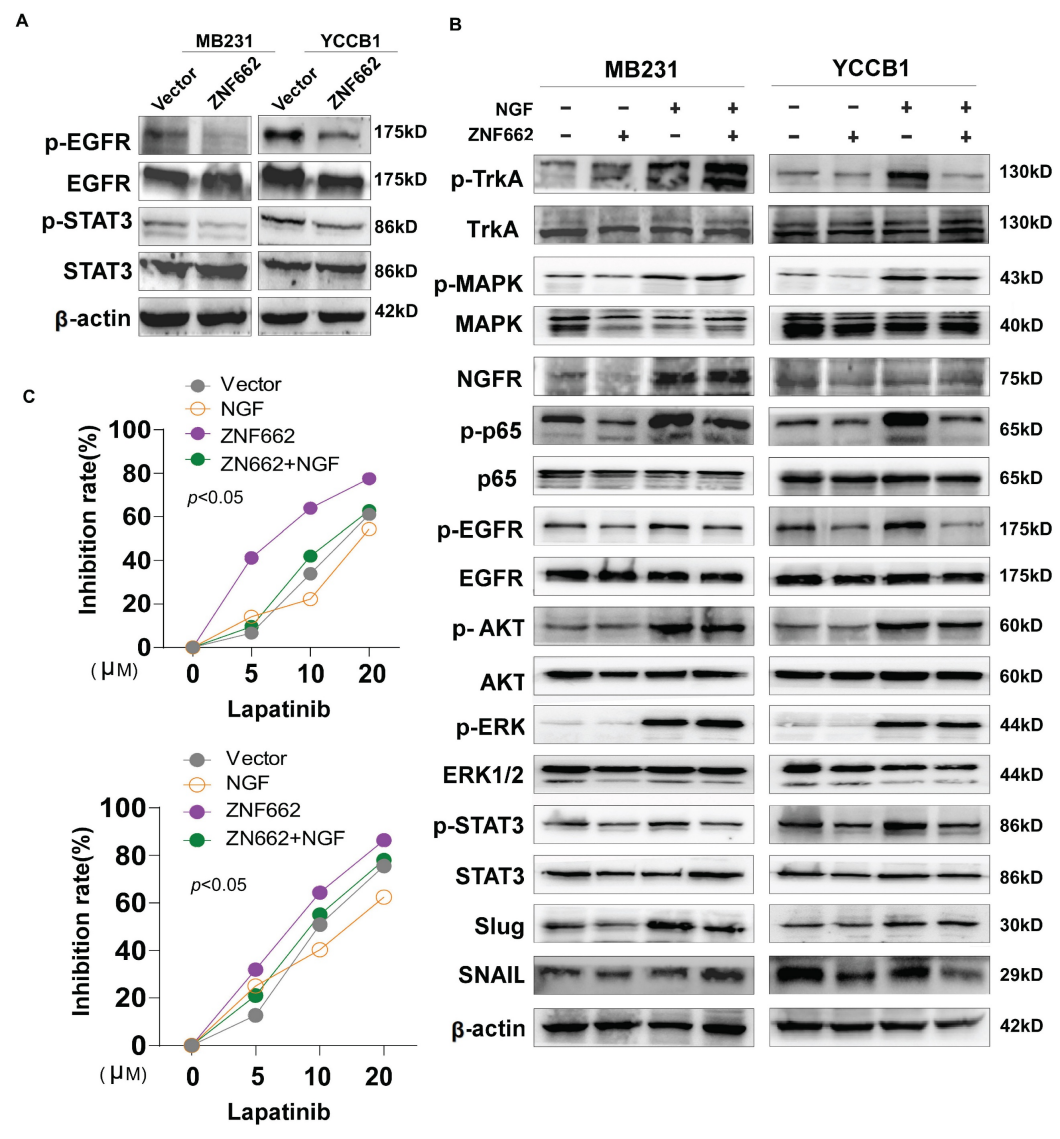
** The ZNF662-NGF axis regulates the PI3K/AKT and STAT3 pathways in TNBC cells, and ZNF662 could increase the sensitivity of TNBC cells to EGFR inhibitor lapatinib.** (A) After over-expression of ZNF662 in MB231 and YCCB1 cells, the protein levels of p-EGFR, EGFR, p-STAT3 and STAT3 were examined by Western blot. (B) Effect of ZNF662 re-expression on downstream signaling pathways was examined by Western blot. (C) Proliferation inhibition rate in ZNF662- or NGF-overexpressing cells treated with different concentrations of lapatinib.

In order to further explore the specific molecular mechanism of ZNF662-NGF axis in TNBC, we conducted the literature search. It was reported that the NGF-TrkA-STAT3 axis could trigger epithelial-mesenchymal transition (EMT) in malignant tumor and confer resistance to the EGFR inhibitor 37. Additionally, NGF was found to be involved in the activation of the PI3K/AKT signaling pathway in breast cancer 34, which was consistent with our enrichment analysis results (Fig. 6D). Therefore, we investigated whether the NGF-STAT3/NGF-PI3K signaling axis could be associated with the tumor suppressor phenotypic change caused by ZNF662. Not surprisingly, ZNF662 could decrease the activity of NGF-STAT3/NGF-PI3K signaling axis by suppressing NGF expression. Moreover, the decreased protein levels of p-EGFR might result from the reduced expression of NGF (Fig. 8A+B). In contrast, p-EGFR and p-AKT protein levels were increased in MB468 cells after ZNF662 knockdown (Additional file 2: sFig. 3E). To further confirm the correlation between ZNF662 and NGF, we transfected ZNF662 plasmid into control and NGF-overexpressing MB231 and YCCB1 cells and performed protein extraction. Results suggested that ZNF662 could reverse the altered protein levels caused by NGF over-expression, indicating its critical role in the regulatory axis (Fig. 8B). Taking these results together, our studies demonstrated that ZNF662-NGF axis was involved in the regulation of the PI3K/AKT and STAT3 pathways in TNBC cells.

### ZNF662 could increase the sensitivity of TNBC cells to EGFR inhibitor lapatinib

As mentioned earlier, the NGF signaling axis was associated with the sensitivity of cancer cells to EGFR inhibitors in malignant tumor. Moreover, targeting EGFR represents a promising therapeutic strategy in TNBC. Thus, we aimed to investigate whether the ZNF662-NGF axis could modulate the sensitivity of TNBC cells to anti-cancer drugs, particularly EGFR inhibitors. Next, we performed an initial screening using our own drug library and discovered several drug candidates. It is worth mentioning that lapatinib exhibited the highest anti-proliferative activity against ZNF662-overexpressing TNBC cells (data not shown). This suggests its dual HER2/EGFR targeting may provide unique therapeutic advantages in TNBC. Other EGFR inhibitors, such as gefitinib, were excluded due to limited efficacy. Additionally, non-EGFR-targeting candidates, such as herbal compounds, were also excluded because of their nonspecific mechanisms and potential toxicity. Finally, we decided to focus on lapatinib for further study. Here, we found that ZNF662-overexpressing TNBC cells had an increased inhibition rate to lapatinib compared to control cells, suggesting they are more sensitive (Fig. 8C). However, the specific mechanisms still need to be studied and further investigated. In conclusion, our results indicated that ZNF662 could regulate the sensitivity of TNBC cells to EGFR inhibitor lapatinib.

### TrkA inhibitor GW441756 could suppress the activation of downstream STAT3 signaling pathway in TNBC cells

**Figure 9:**
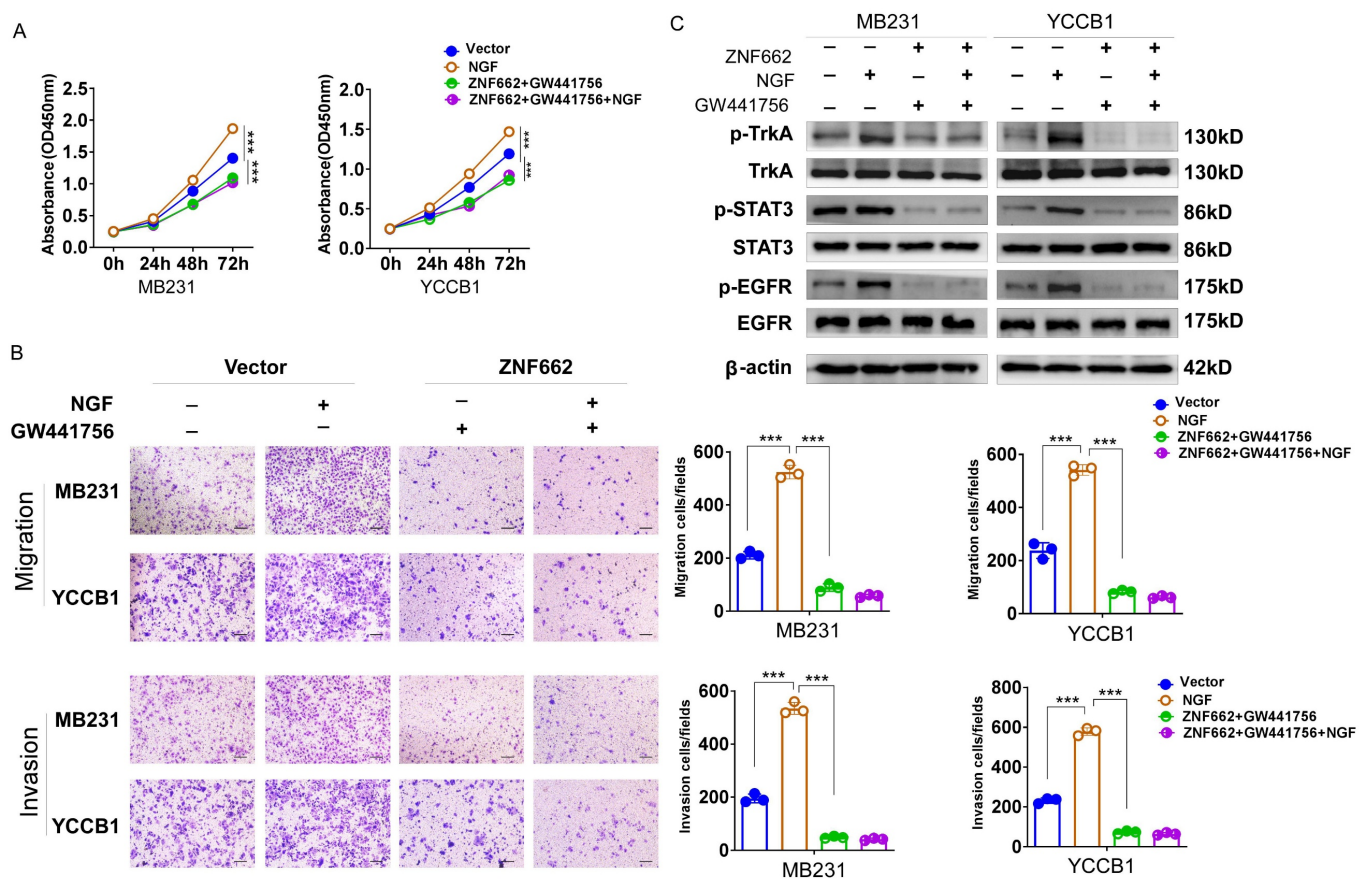
** TrkA inhibitor GW441756 could affect ZNF662-NGF axis in TNBC cells.** (A) Effect of GW441756 on MB231 and YCCB1 cell proliferation was determined by CCK-8 assay. (B) Effect of GW441756 on MB231 and YCCB1 cell migration and invasion was determined by Transwell assay. (C) Effect of GW441756 on downstream signaling pathways was examined by Western blot. GW441756 was purchased from MCE (HY-18314, Shanghai, China) and used at a concentration of 1 μM. *p*-value was assessed by Student's t-test. Scale bars: 100 μm. All data are presented as the mean ± SD. SD, standard deviation. *p < 0.05, **p < 0.01, ***p < 0.001.

To further verify the crucial role of ZNF662-NGF axis in TNBC progression, we used a potent and specific TrkA inhibitor GW441756 (targeting NGF receptor) to effectively block downstream pathways. CCK-8 and Transwell assays were conducted to examine the effect of GW441756 on TNBC cell proliferation and metastasis. Results suggested that GW441756 was able to reverse the oncogenic effect of NGF in TNBC cells (Fig. 9A+B). In addition, western blot also confirmed that the downstream STAT3 signaling pathway was affected by GW441756 (Fig. 9C), suggesting ZNF662-NGF axis might be a potential therapeutic target in TNBC. Altogether, these results indicated that TrkA inhibitor GW441756 could affect ZNF662-NGF axis in TNBC.

## Discussion

Breast cancer, especially TNBC, has become one of the most fatal breast diseases threatening the health of women worldwide. Its characteristics, such as strong invasiveness, poor prognosis, and lack of targeted therapy, make it urgent to identify effective tumor biomarkers for early diagnosis and treatment 38.

KRAB-ZFPs refer to a family of ZFPs containing Krüppel-associated box (KRAB) conserved domains, most of which are potent transcriptional repressors. The N-terminal KRAB domain plays a critical role in transcriptional inhibition by interacting with the corepressor, while the main function of C-terminal zinc finger domain is to bind specific DNA sequences 39, 40. As mentioned in the Introduction, an increasing number of studies have revealed the relationship between dysregulation of KRAB-ZFPs family and breast cancer progression. For example, ZFP57 was found to play a tumor suppressor role through inhibiting Wnt/β-catenin signaling pathway 41. Our previous work had elucidated that ZNF334 restrained the proliferation and metastasis ability of TNBC cells by regulating SFRP1-Wnt/β-catenin signaling axis 29.

As a novel member of the KRAB-ZFPs family, ZNF662 was identified. Previous research demonstrated that ZNF662 was down-regulated due to DNA methylation in oral squamous cell carcinoma and could be used as a prognostic marker 25. However, how ZNF662 influences tumor progression remains unclear. In this study, we confirmed the tumor-suppressive effect of ZNF662 in TNBC and revealed the underlying mechanism. We found that ZNF662 was lowly expressed in breast cancer tissues and cells. Online database analysis revealed that higher expression of ZNF662 is associated with better prognosis of breast cancer patients. We further demonstrated that the promoter hyper-methylation of ZNF662 was the main cause of its down-regulation in breast cancer. Over-expression of ZNF662 could inhibit proliferation and metastasis, as well as induce cell cycle arrest in TNBC cells. In addition, ZNF662 hindered the growth and metastasis of xenograft tumors *in vivo*. In conclusion, ZNF662 might be a new tumor suppressor in TNBC.

To further explore the underlying mechanism of ZNF662 in TNBC, we performed high-throughput RNA sequencing. Then, we screened differentially expressed genes and finally focused on the NGF gene. Nerve growth factor (NGF) is one of the most important members of the neurotrophic factor family. It could bind to two specific membrane receptors: one is the ligand-specific tropomyosin receptor kinase A (TrkA), and the other is the p75/NGFR 42, 43. More importantly, the relationship between NGF and tumorigenesis has attracted increasing attention in the recent years. In terms of breast cancer, it had been reported that primary breast tumor cells can acquire NGF to promote their survival, proliferation and metastasis in an autocrine/paracrine manner 44-46. Moreover, NGF had been previously reported to promote tumorigenesis in TNBC by activating the PI3K and STAT3 signaling pathways 47, 48. It was worth mentioning that NGF signaling axis had been proven to be involved in resistance to chemotherapy in breast cancer 49-51. Therefore, targeting the NGF signaling axis may be a promising treatment strategy for breast cancer patients.

Next, we confirmed that ZNF662 could directly bind to the NGF promoter sites and inhibit its transcription activity, and further demonstrated ZNF662 could decrease the activity of NGF-STAT3/NGF-PI3K signaling axis by suppressing NGF expression in TNBC. Collectively, our results showed that ZNF662 functioned as a tumor suppressor in TNBC through suppressing NGF signaling axis.

Searching for effective therapeutic targets of TNBC might further contribute to the development of TNBC treatment approaches. In terms of therapeutic efficacy of EGFR-TKI, although EGFR is frequently over-expressed in more than 70% of TNBC patients, the clinical application of EGFR inhibitors in TNBC is still limited 6. Previous studies reported that NGF-TrkA axis could confer resistance to EGFR inhibitors in malignant tumor 37. From this perspective, we found that ZNF662-NGF axis could regulate the sensitivity of TNBC cells to EGFR inhibitor lapatinib. Interestingly, lapatinib, an EGFR and HER2+ receptor inhibitor, is broadly used for treatment of HER2+ advanced breast cancer patients 52. However, there is still insufficient evidence on the effectiveness of lapatinib for the TNBC treatment due to the lack of predictive biomarkers. Thus, we considered whether we could combine lapatinib with other chemotherapeutic agents to manage TNBC after detecting the expression or methylation status of ZNF662 in tumor samples. In addition, it remains unclear whether the combination of NGF-TrkA axis inhibitor with lapatinib can provide superior outcomes for TNBC patients. Therefore, further in-depth investigations are still required. Moreover, there are still many problems that must be overcome from basic research to clinical application, which is also a deficiency of our research. All in all, we conclude that ZNF662 could become a potential therapeutic target for TNBC.

## Conclusions

**Figure 10:**
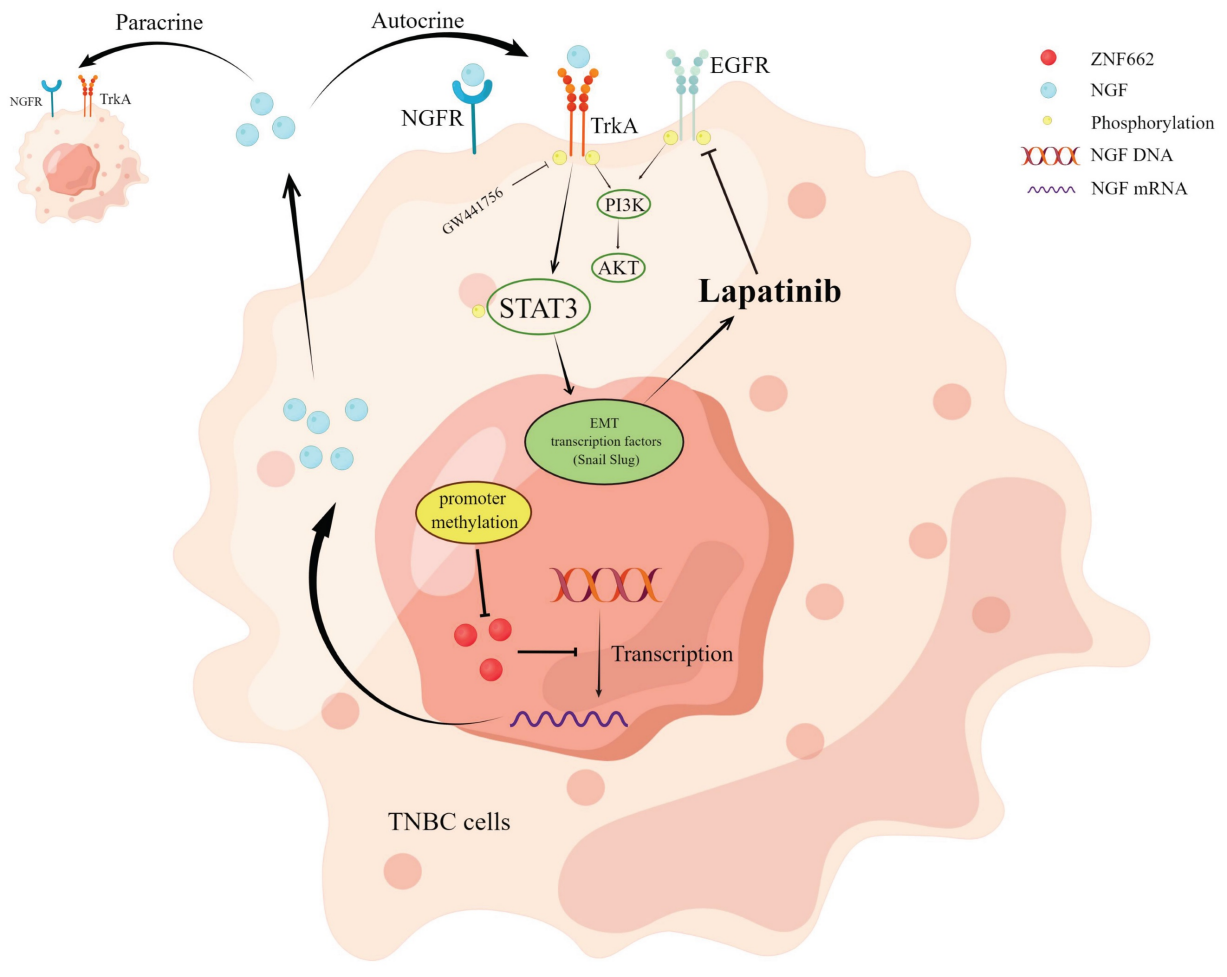
Molecular mechanism of ZNF662-NGF axis inhibiting TNBC.

Our study identifies ZNF662 as a novel member of KRAB-ZFPs family in TNBC. ZNF662 acts as an important tumor suppressor gene in TNBC but is often down-regulated due to promoter hyper-methylation. ZNF662 affects the PI3K/AKT and STAT3 signaling pathways by down-regulating NGF expression, thereby inhibiting the occurrence and development of TNBC (Fig. 10). In summary, ZNF662 may become a potential biomarker for screening, diagnosis and treatment of TNBC.

## Supplementary Material

Supplementary methods, figures and table.

## Abbreviations

ZFPs: Zinc Finger Proteins
KRAB-ZFPs: KRAB-containing zinc finger proteins
ZNF662: Zinc Finger Protein 662
TNBC: Triple-negative breast cancer
MSP: Methylation-specific PCR
Aza: 5-aza-2′-deoxycytidine
TSA: Trichostatin A
IHC: Immunohistochemistry
ChIP: Chromatin Immunoprecipitation
EMT: Epithelial-mesenchymal transition
NGF: Nerve growth factor
TrkA: Tropomyosin receptor kinase A
EGFR: Epidermal growth factor receptor
RTK: Receptor tyrosine kinase
