# Supplementary material for: Promoter Hyper-methylation of ZNF662 Restrains its Tumor Suppressing Function in Triple-Negative Breast Cancer Through Regulating NGF Signaling Axis
Source: Int J Biol Sci. 2025 Jun 12;21(9):4081–97. doi: 10.7150/ijbs.102940 (PMC12223779; doi:10.7150/ijbs.102940)
Supplement: Supplementary file 1 — Supplementary methods, figures and table. [file ijbsv21p4081s1.pdf]

Additional file

Additional file 1: Table S1. List of primers used in this study

Additional file 2: sFig. 1 Methylation status of ZNF662 in BRCA. (A) Correlation between ZNF662 methylation status and ZNF662 mRNA expression in BRCA from cBioPortal database. (B) Part of the cluster of methylated CpG sites of ZNF662 in BRCA from MethSurv database. Red: hyper-methylation. (C) The prognostic values of methylation-associated CpG sites cg04146745, cg26523005 and cg18356403 in BRCA from MethSurv database.

sFig. 2 Aza treatment could impair malignant biological phenotypes of TNBC cells. (A) CCK-8 assay revealed the effects of Aza treatment on the proliferation of TNBC cells. (B-C) Transwell assays revealed the effects of Aza treatment on the migration of TNBC cells. (D-E) Transwell assays revealed the effects of Aza treatment on the invasion of TNBC cells.

sFig. 3 Knockdown of ZNF662 regulates the PI3K/AKT pathway in MB468 cells. (A+B) Validation of ZNF662 knockdown efficiency by qRT-PCR and Western blot. (C) Colony formation assay in siNC- and siZNF662-transfected MB468 cells. (D) Cell cycle distribution of siNC- and siZNF662-transfected MB468 cells. (E) After ZNF662 knockdown in MB468 cells, expression of downstream protein levels was examined by Western blot. All data are shown as mean ± SD from three independent experiments. SD, standard deviation. ***$p < 0.001$.

Table. S1

Primer		Sequence (5'-3')	Product Size
RT-PCR/ qRT-PCR	ZNF662-F	GACCTCATGGTCCTATCAAGTG	181bp
	ZNF662-R	CTACTGAGATCATCTCATCCTTG	
	NGF-F	CACACTCAGAGAGCAATGTCC	132bp
	NGF-R	CCACGCGTGCAGCTATCGC	
	β-actin-F1	GTCTTCCCCTCCATCGTG	113bp
	β-actin-R1	AGGGTGAGGATGCCTCTCTT	
Chip-PCR	NGFproF1	TTGTCAGAGGAGCTTGGGCAC	199bp
	NGFproR1	TTGCTCGCAGCTTCCCTCAC	
MSP	ZNF662-m1	GTGGAGTCGGGGTTTTATTTC	131bp
	ZNF662-m2	CCTCTAAAAAAACGAAAACGCG	
	ZNF662-u1	GAGTGGAGTTGGGGTTTTATTTT	136bp
	ZNF662-u2	TCCCTCTAAAAAAACAAAAACACA	

sFig.1

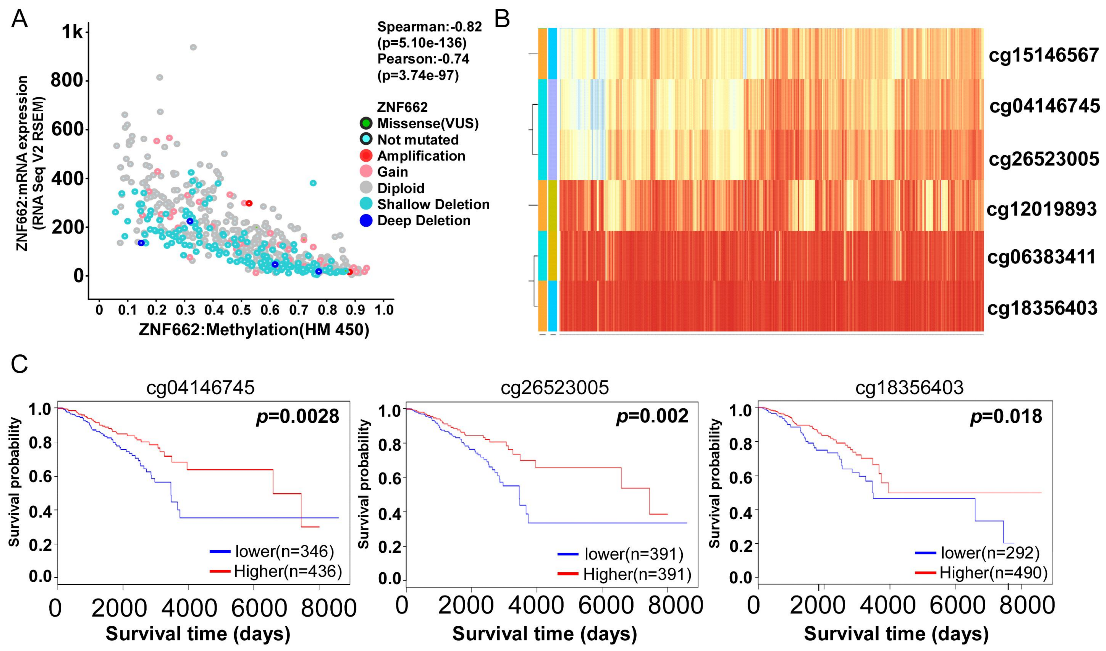
A
B
C
Spearman:-0.82
(p=5.10e-136)
Pearson:-0.74
(p=3.74e-97)
ZNF662
Missense(VUS)
Not mutated
Amplification
Gain
Diploid
Shallow Deletion
Deep Deletion
ZNF662:mRNA expression (RNA Seq V2 RSEM)
ZNF662:Methylation(HM 450)
cg15146567
cg04146745
cg26523005
cg12019893
cg06383411
cg18356403
cg04146745
p=0.0028
lower(n=346)
Higher(n=436)
cg26523005
p=0.002
lower(n=391)
Higher(n=391)
cg18356403
p=0.018
lower(n=292)
Higher(n=490)
Survival probability
Survival time (days)

sFig.2

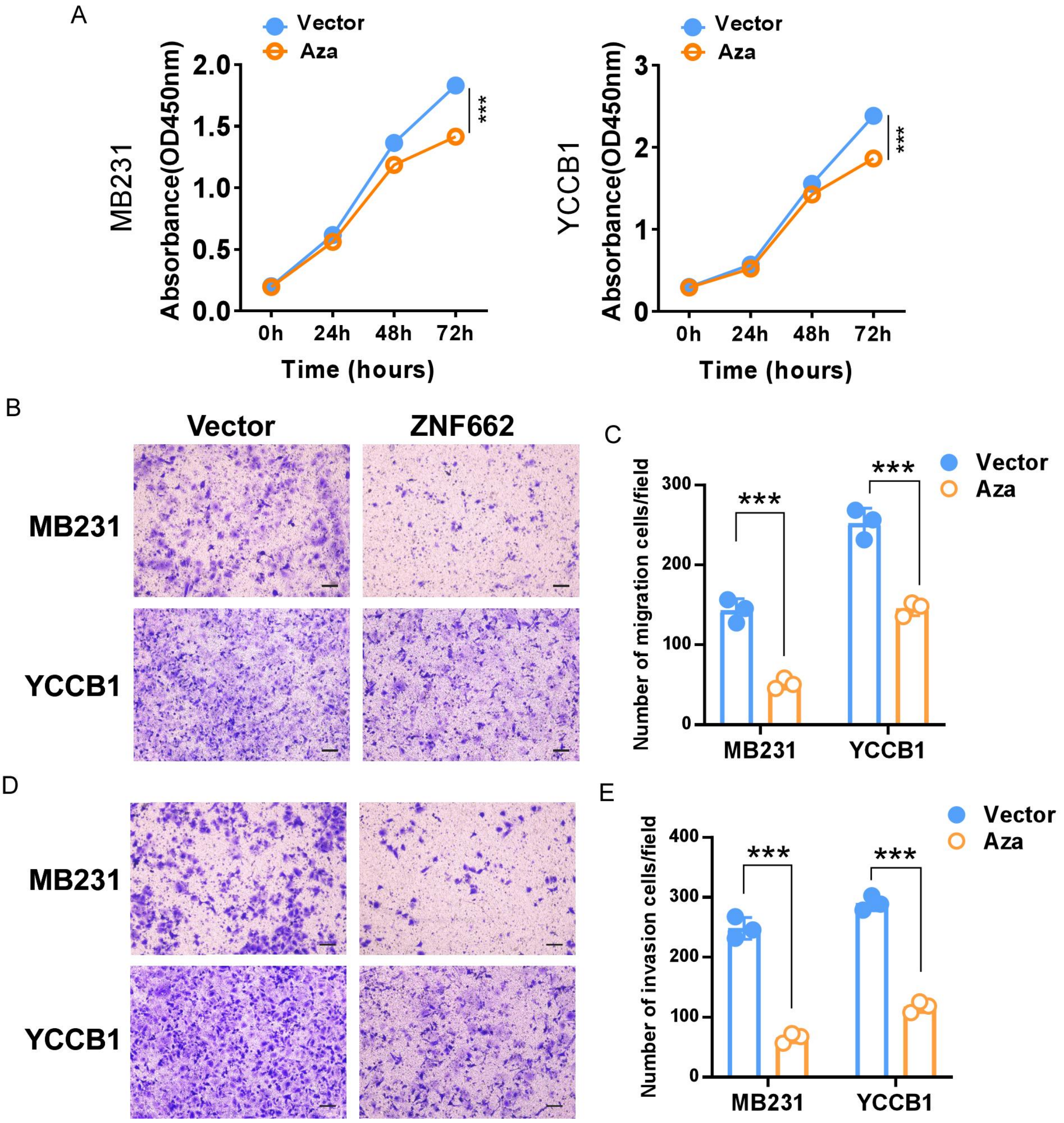
A
Vector
Aza
MB231
Absorbance(OD450nm)
2.0
1.5
1.0
0.5
0.0
***
0h
24h
48h
72h
Time (hours)
YCCB1
Absorbance(OD450nm)
3
2
1
0
B
Vector
ZNF662
MB231
YCCB1
C
Number of migration cells/field
300
200
100
0
MB231
YCCB1
D
E
Number of invasion cells/field
400

sFig. 3

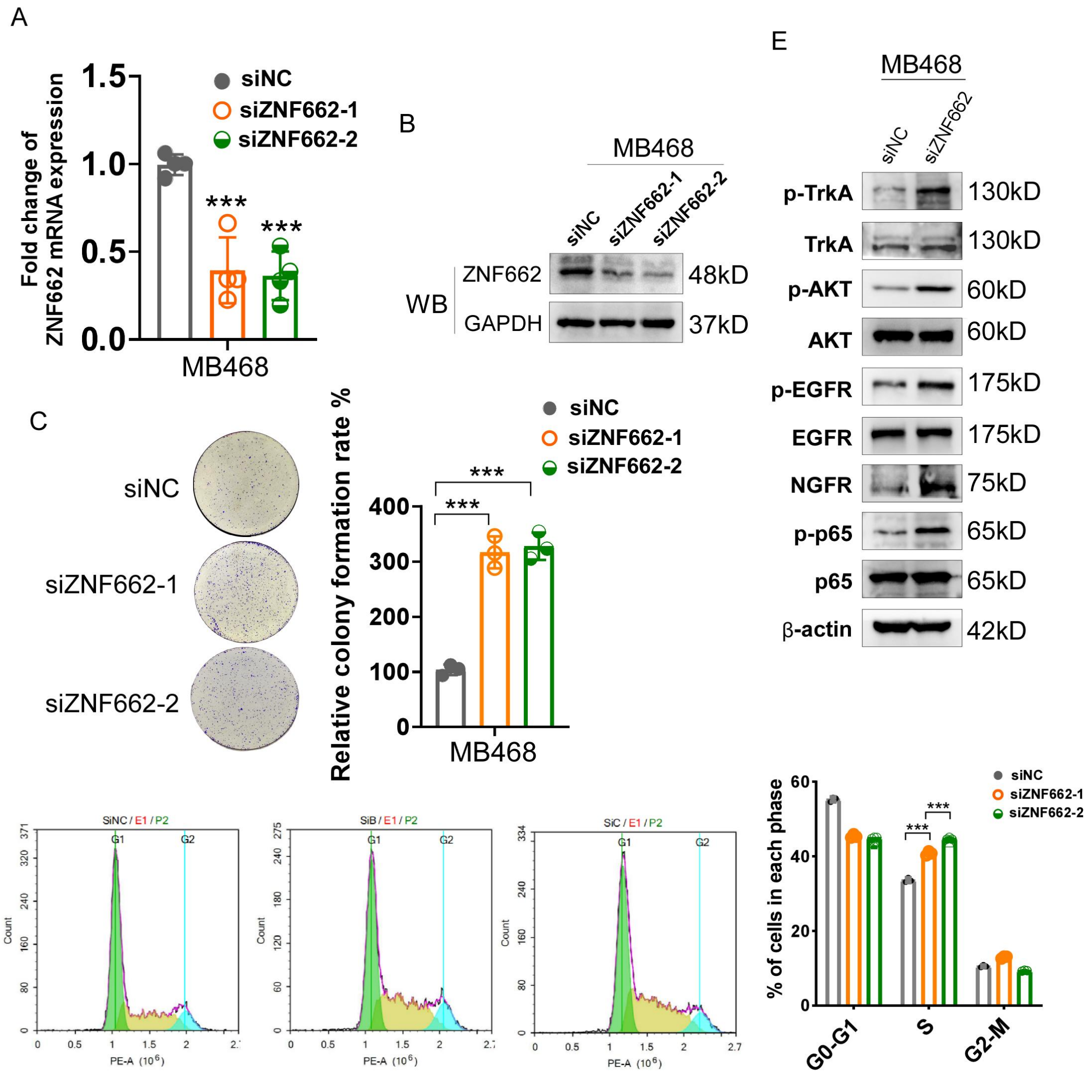
A
siNC
siZNF662-1
siZNF662-2
Fold change of ZNF662 mRNA expression
1.5
1.0
0.5
0.0
***
***
MB468
B
MB468
siNC
siZNF662-1
siZNF662-2
WB
ZNF662
GAPDH
48kD
37kD
E
MB468
siNC
siZNF662
p-TrkA 130kD
TrkA 130kD
p-AKT 60kD
AKT 60kD
p-EGFR 175kD
EGFR 175kD
NGFR 75kD
p-p65 65kD
p65 65kD
β-actin 42kD
C
siNC
siZNF662-1
siZNF662-2
Relative colony formation rate %
400
300
200
100
0
***
***
MB468
D
SiNC / E1 / P2
SiB / E1 / P2
SiC / E1 / P2
G1
G2
Count
PE-A (10^6)
% of cells in each phase
60
40
20
0
G0-G1
S
G2-M
***
***
